# Hysteresis in Center of Mass Velocity Control during the Stance Phase of Treadmill Walking

**DOI:** 10.3389/fnhum.2017.00187

**Published:** 2017-04-27

**Authors:** Kyoung-Hyun Lee, Raymond K. Chong

**Affiliations:** ^1^Center for Sport Science in Gwangju, Gwangju Sports CouncilGwangju, South Korea; ^2^Department of Physical Therapy, Augusta UniversityAugusta, GA, USA

**Keywords:** balance control, dynamical systems, gait, human, postural control

## Abstract

Achieving a soft landing during walking can be quantified by analyzing changes in the vertical velocity of the body center of mass (CoM) just prior to the landing of the swing limb. Previous research suggests that walking speed and step length may predictably influence the extent of this CoM control. Here we ask how stable this control is. We altered treadmill walking speed by systematically increasing or decreasing it at fixed intervals. We then reversed direction. We hypothesized that the control of the CoM vertical velocity during the late stance of the walking gait may serve as an order parameter which has an attribute of hysteresis. The presence of hysteresis implies that the CoM control is not based on simply knowing the current input conditions to predict the output response. Instead, there is also the influence of previous speed conditions on the ongoing responses. We found that the magnitudes of CoM control were different depending on whether the treadmill speed (as the control parameter) was ramped up or down. Changes in step length also influenced CoM control. A stronger effect was observed when the treadmill speed was speeded up compared to down. However, the effect of speed direction remained significant after controlling for step length. The hysteresis effect of CoM control as a function of speed history demonstrated in the current study suggests that the regulation of CoM vertical velocity during late stance is influenced by previous external conditions and constraints which combine to influence the desired behavioral outcome.

## Introduction

During walking, the body center of mass (CoM) moves in three dimensions. Along the sagittal plane, it can be described as oscillating up and down. It is displaced at its highest point at the mid-stance phase and lowest at the double-support phase. The primary goal is to move the body forward from one location to another without losing control of balance or falling. Neuromuscular control comprises the coordination of muscle contractions which occur at the right time, amplitude and duration. Muscles are activated to modulate the landing of the swing limb, progress the body and manage inter-limb coordination (Olree and Vaughan, 1995). Muscle activities also influence the stiffness of connective tissues surrounding the joints of the body (Ferris et al., 1999).

As the CoM is displaced downwards during late swing, it will free-fall if muscles are not adequately activated to soften the landing (Brenière and Bril, 1998). Free-falling typically occurs in other gaits which have a flight phase such as running, hopping and jumping. In these gaits, once the body takes off in the air, gravity takes over. There is no way to control the CoM trajectory, acceleration or final speed of the subsequent landing (Farley and Ferris, 1998). Free falling of the swing limb can also happen in walking robots if designers do not adequately consider how the landing limb is controlled (Collins et al., 2005).

Although the swing limb is not passively oscillating as it transitions to the stance phase (Whittlesey et al., 2000), controlling the falling CoM must be accomplished by the contralateral stance limb. Evidence of such control comes from the observation of a reduction (i.e., braking) in the CoM vertical velocity before foot contact of the swing limb (Brenière and Bril, 1998). At shorter or slower than normal steps, braking the CoM is minimal. The mechanical contact of the swing limb with the floor contributes to stopping the CoM from further falling. At longer and faster steps, braking the CoM increases, reflecting increasing control of the falling CoM (Brenière and Bril, 1998; Chong et al., 2009).

These observations were obtained from a randomized order of walking under different step lengths and speeds. The assumption was that knowing the current gait parameters and how they are adjusted based on the imposition of walking speed is enough for ample prediction of the walking behavior (Brenière, 2003). In the present study, we systematically increased and decreased walking speeds, the control parameter, in healthy young adults to test the hypothesis that the braking of the CoM may also be affected by prior gait conditions, i.e., there is a history effect which should be accounted for as well. Based on the dynamical systems approach to the study of interlimb coordination (Kelso et al., 1981), we ask whether CoM control may serve as a novel order parameter which has an attribute of hysteresis as a reflection of the history effect. Evidence of hysteresis may suggest that the degree of CoM braking at a given walking speed cannot all be attributed to current conditions. Therefore, CoM control cannot be predicted simply based on a linear and stable input-output response. It would instead be a semi-open system that is compelled to self-organize from a range of flexible non-linear spatiotemporal gait patterns (Kelso, 1995). Such a trial order-like history effect would suggest that CoM control can be better ascertained by considering immediate prior conditions such as whether the individual is speeding up or slowing down during walking.

## Materials and Methods

### Participants

A convenience sample of six healthy young adults (three men and three women, 25 ± 2 years old) participated in the study which was approved by the Augusta University institutional review board. The experiment was carried out with the understanding and written consent of each subject. Subjects wore shorts or swim trunks (plus sports bra for the women) and flat shoes during the experiment.

### Procedures

The experiment involved two treadmill speed conditions. In the Ramp-up condition, subjects started at a low speed and walked at increasingly faster speeds without taking any break in between speed changes. In the Ramp-down condition, subjects started at a high speed and eventually slowed down. The speeds of the treadmill were standardized by first obtaining the preferred walking speed of each subject (P) following several minutes of familiarization on the treadmill (Arsenault et al., 1986). Values for 11 speed increments were then calculated in 0.133 m/s intervals: three speeds slower (P − 1 to P − 3) and eight speeds faster (P + 1 to P + 8) than the preferred speed of each subject. Subjects walked 10 steps at each treadmill speed. Incremental changes in treadmill speeds occurred on every 11th step. Subjects then continued walking the next 10 steps at the new speed, and so on until they completed their pre-determined P − 3 or P + 8 speeds. The test order of the Ramp-up and Ramp-down conditions were counterbalanced among the subjects. In between the test conditions, subjects dismounted from the treadmill and rested between 10–15 min.

### Data Reduction

**Figure 1:**
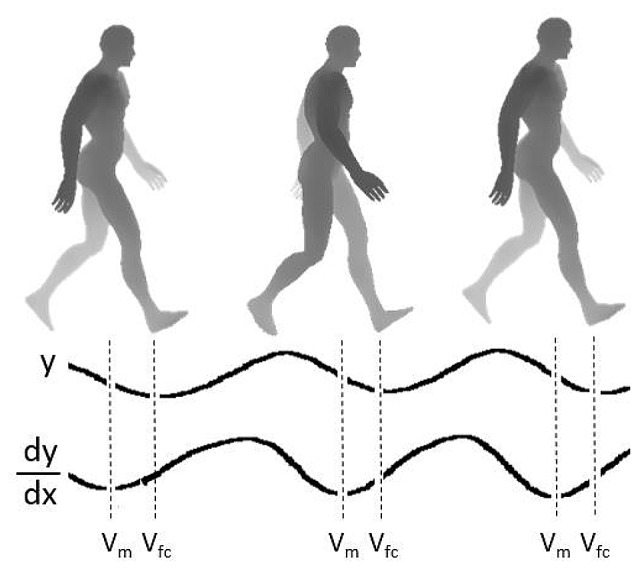
**Illustration of the rising and falling of the body center of mass (CoM) during the walking gait.** y = vertical displacement, dy/dx = vertical velocity, V_m_ = maximum CoM vertical velocity occurring between mid- to late stance, V_fc_ = CoM vertical velocity at foot contact. The braking index is calculated as follows: braking index = V_m_ − V_fc_/V_m_. The index that is obtained from this ratio is a dimensionless number ranging between 0 and 1. The index will have a small value if there is little braking before foot contact, i.e., V_fc_ is similar to V_m_. Conversely, if V_fc_ is small, the index will have a large value indicating the presence of “braking.”

The amount of braking of the CoM velocity during late stance was quantified as follows (Brenière and Bril, 1998; Chong et al., 2009):

$${\text{braking index = V}}_{\text{m}}\text{− }{\text{V}}_{\text{fc}}/{\text{V}}_{\text{m}},$$

where V_m_ = maximum CoM vertical velocity occurring between mid- to late stance, and V_fc_ = vertical velocity of CoM at foot contact (Figure 1).

The index that is obtained from this ratio is a dimensionless number. It indicates the amount of braking in CoM vertical velocity at foot contact relative to its maximum value. V_fc_ will be similar to V_m_ if there is only a small amount of braking, resulting in a small index approaching zero. Conversely, if V_fc_ has a small value, the index will have a large value approaching a maximal value of 1 indicating that there is a substantial braking of the CoM velocity.

The CoM was derived from a 7-segment model (foot, leg, thigh and head-arm-trunk, HAT) using the 6-camera 3D PEAK Motus motion capture system (120 Hz). Raw marker coordinate data were low-pass filtered with a dual pass 4th order Butterworth filter and a 6 Hz cutoff frequency.

Step length was estimated by taking the antero-posterior distance between the heel marker of the leading foot at the instance of foot contact (before the marker starts to displace posteriorly) and the heel marker of the trailing foot.

Each subject’s walking gait was inspected to ensure that there was no flight phase at his/her highest treadmill speed which, if present, indicated that the subject broke into a run and confounded the experiment (Raynor et al., 2002; Hreljac et al., 2007).

### Analyses

For every speed interval of the Ramp-up and Ramp-down condition, each subject’s 10 steps of braking index and step length were averaged. The 11th step was a perturbation step (due to the change in treadmill speed) and was not included in the analyses. The averaged values were then combined with the other subjects to obtain the group average. These values at the preferred speed were compared to their corresponding values during the Ramp-up and down conditions using paired *t*-tests. The threshold for significance was Bonferroni-adjusted at *p* < 0.025. Initial analyses revealed that subjects changed their step length as the treadmill speed was ramped up or down. In order to determine the independent effects of step length, separate analyses of covariance were conducted (using the PROC REG command of the SAS statistical analysis software) to determine whether the braking index differed as a function of step length. The threshold for statistical significance was set at *p* < 0.05. The covariance analyses were followed by the two-sample Kolmogorov-Smirnov test (KS-test). If the braking index demonstrates hysteresis, the KS-test should be significant, meaning that the distribution of the braking index data across the range of treadmill speeds is different when the treadmill was speeded up vs. down.

## Results

The average preferred walking speed of the subjects was 1.09 ± 0.11 m/s. The average maximum and minimum walking speeds of the subjects were 2.16 ± 0.11 m/s and 0.69 ± 0.11 m/s, respectively. None of the subjects broke into a run when walking at high speeds.

**Figure 2:**
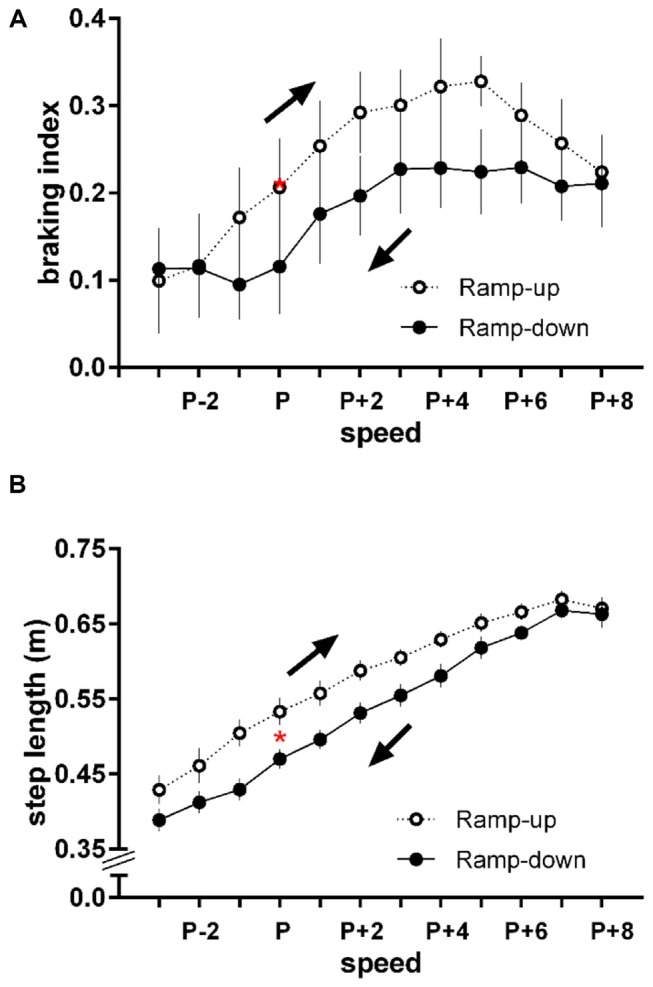
**Changes in (A)** braking index and **(B)** step length (mean ± standard error) as a function of direction of change in speed. Arrows indicate the direction of the change in speed. P = preferred walking speed of each subject; P − 1 to P − 3 = slower speed intervals; P + 1 to P + 8 = faster speed intervals. Speed intervals were standardized in 0.133 m/s increments relative to each subject’s preferred speed. Red asterisks (*) indicate the average braking index and step length values at the preferred walking speed: *p* > 0.05 compared to the Ramp-up condition at the same speed; *p* = 0.003 and *p* = 0.002, respectively, compared to the Ramp-down condition. The average preferred walking speed of the subjects was 1.09 ± 0.11 m/s. Average maximum and minimum walking speeds were 2.16 ± 0.11 m/s and 0.69 ± 0.11 m/s, respectively. No subject broke into a run at their highest speed. Refer to Figures 3A,B for additional analyses of the braking index.

The braking index at the preferred speed was 0.207 ± 0.132 compared to 0.207 ± 0.138 during the Ramp-up condition (*p* > 0.05) and 0.116 ± 0.135 during the Ramp-down condition (*p* = 0.003, Figure 2A). The step length at the preferred speed was 0.51 ± 0.03 m compared to 0.53 ± 0.04 m during the Ramp-up condition (*p* > 0.05) and 0.47 ± 0.03 m during the Ramp-down condition (*p* = 0.002, Figure 2B).

### Analysis of Covariance

#### Ramp-Up Condition

The correlation between the braking index and step length was 0.58 (*p* < 0.0001). The regression portion of the analysis was significant for step length but not treadmill speed, *F*_(1,11)_ = 15.43, *p =* 0.0002, *r*^2^ = 0.34 and *F*_(1,11)_ = 1.15, *p =* 0.34, *r*^2^ = 0.18, respectively, indicating that step length but not treadmill speed was significantly related to the braking index.

#### Ramp-Down Condition

The correlation between the braking index and step length was 0.54 (*p* < 0.0001). The regression portion of the analysis was significant for step length, *F*_(1,11)_ = 33.80, *p =* 0.0001, *r*^2^ = 0.29, indicating that step length was significantly related to the braking index.

After accounting for the effects of step length, the treatment effect of treadmill speed remained significant, *F*_(1,11)_ = 1.15, *p =* 0.039, *r*^2^ = 0.28.

### Test of Hysteresis

#### Braking Index

The mean for a normal distribution of the braking index in the Ramp-up condition was 0.23 ± 0.09, *p* = 0.85. For the Ramp-down condition, the mean was 0.18 ± 0.06, *p* = 0.57 (Table 1A).

**Table 1:** **Results of the Kolmogorov-Smirnov test of normal distribution for the Ramp-up and Ramp-down speed conditions for the (A) braking index and (B) step length**.

	Ramp-Up condition	Ramp-Down condition
**(A) Braking index**		
Mean	0.24	0.18
95% Confidence interval	0.19–0.29	0.14–0.21
Standard deviation	0.07	0.05
High and low	0.33, 0.10	0.23, 0.1
3rd Quartile	0.30	0.27
1st Quartile	0.18	0.12
Median	0.26	0.20
Average absolute deviation from median	0.06	0.04
Test of normal distribution of data	*p* = 0.85	*p* = 0.57
**(B) Step length (meters)**		
Mean	0.58	0.54
95% Confidence interval	0.53–0.64	0.48–0.60
Standard deviation	0.08	0.01
High and low	0.68, 0.43	0.67, 0.39
3rd Quartile	0.66	0.63
1st Quartile	0.51	0.44
Median	0.60	0.54
Average absolute deviation from median	0.07	0.08
Test of normal distribution of data	*p* = 0.94	*p* = 0.99

**Figure 3:**
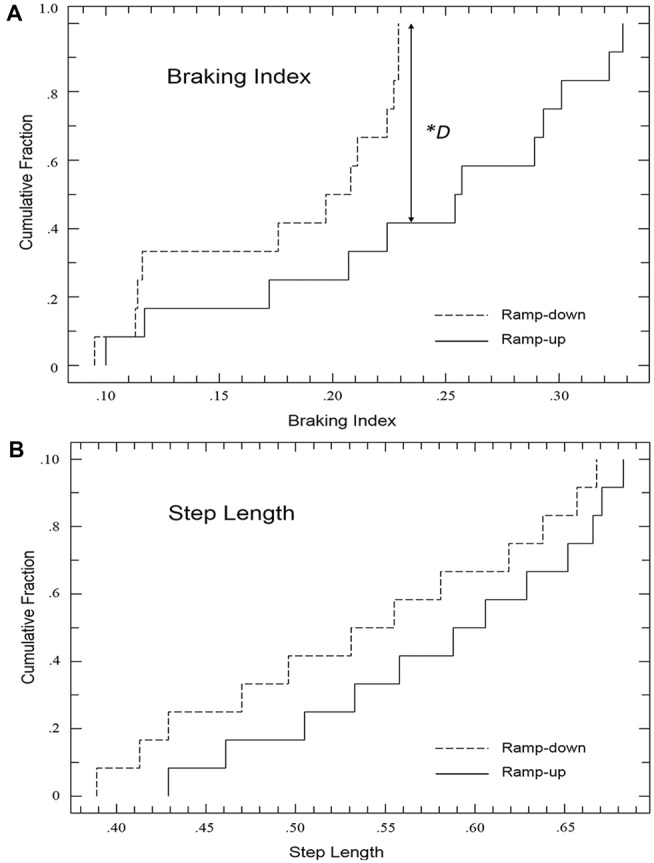
**KS-Test comparison cumulative fraction plots for the (A)** braking index and** (B)** step length. *The maximum difference between the cumulative distributions of the braking index is significant, *D* = 0.58, *p* = 0.019. This corresponds to the speed interval of P + 5 (*p* = 0.027, Figure 1). The maximum difference between the cumulative distributions of the braking index is not significant, *D* = 0.25, *p* = 0.79.

The KS-test revealed that the distribution of the datasets between the two conditions were different. The maximum difference between the cumulative distributions of the braking index as the treadmill speed was ramped up vs. down is *D* = 0.58 with a corresponding *p* value of 0.019 (Figure 3A). The treadmill speed in which the largest difference in braking index value occurred was P + 5 (*p* = 0.027, Figure 2A).

#### Step Length

The mean for a normal distribution of step length in the Ramp-up condition was 0.58 ± 0.10 m, *p* = 0.94. For the Ramp-down condition, the mean was 0.54 ± 0.11 m, *p* = 0.99 (Table 1B).

The KS-test revealed that the distribution of the datasets between the two conditions were not different, suggesting that the hysteresis effect in step length is not robust. The maximum difference between the cumulative distributions of the braking index as the treadmill speed was ramped up vs. down is *D* = 0.25 with a corresponding *p* value of 0.79 (Figure 3B).

## Discussion

The results of the current study confirmed our hypothesis that CoM velocity braking during the late stance of the walking gait may serve as a novel order parameter that is influenced by the direction of speed changes and therefore displays a hysteresis effect. The degree of CoM braking as a function of treadmill speed is somewhat similar to that obtained during overground walking (Chong et al., 2009). In that study, healthy adults were asked to adjust their step lengths in random order. CoM braking increased gradually as a function of step length before leveling off at longer steps. This pattern of control was also observed in the current study. Differences in the absolute values of the CoM braking index could be due to the treadmill vs. overground walking conditions used in this and the previous Chong study, respectively. Generally however, it is thought that overground and treadmill walking produce similar mechanical and neuromuscular outputs (van Ingen Schenau, 1980; Murray et al., 1985; Taves et al., 1985; Arsenault et al., 1986).

The magnitude of CoM braking is diminished in healthy older adults. In healthy young adults, the CoM braking increased during dual-tasking while step length and walking speed were preserved (Chong et al., 2009). In theory, the degree of braking is not expected to be maximal in which CoM vertical velocity is reduced to zero at foot contact. About two-thirds of the energy required to displace the CoM upward and forward between foot contact and mid-stance is conserved by the exchange between kinetic and potential energies. Their out-of-phase relationship throughout the walking gait cycle is thought to result in this efficient energy conservation (Cavagna et al., 1963). Reducing CoM vertical velocity at foot contact to zero will likely diminish the efficiency of this mechanism.

Other than simply achieving a soft landing, there may be other physiological significances of the CoM braking during the late stance phase of walking that are unknown. Braking of the CoM is thought to represent the integrity of the postural control system (Hahn and Chou, 2003; Michel and Chong, 2004). Toddlers do not exhibit braking until they are 5–6 years old (Brenière and Bril, 1998). Compared to young adults, older adults showed a smaller braking index at the same step length and speed, suggesting an age-related decline in CoM braking (Chong et al., 2009).

Recent studies have also started to shed some light in partial support of the postural control concept. Proprioceptive afferents are thought to be important in producing soleus muscle activities during late stance (Grey et al., 2004), possibly to control stiffness at the ankle (van Jaarsveld et al., 1990; Hansen et al., 2004). Stiffness control however, does not appear be used for controlling walking speed or step length. The increase in muscle activities of the triceps surae with faster walking speed but no corresponding increase in muscle activities when body weight was increased strongly suggests that CoM braking is involved in postural control, i.e., body support rather than gait (forward progression) *per se* (Honeine et al., 2013).

In patients with progressive supranuclear palsy, a neurological condition that presents with significant postural instability in the early stage of the disease, CoM braking was absent during gait initiation (Welter et al., 2007). In patients with Parkinson’s disease (PD), which is another neurological disorder that manifests in postural instability in the later stages of the disease (Cho et al., 2010), 69% showed no CoM braking when their medications were withheld. Less than one-fifth of the patients improved their CoM braking with medication (Chastan et al., 2009a). These two studies seem to suggest that non-dopaminergic pathways may play a critical role in providing adequate CoM braking. In support of this hypothesis, a recent study showed that electrical stimulation of the subthalamic nucleus or substantia nigra pars reticulata but not levodopa-replacement therapy improved CoM braking in PD patients (Chastan et al., 2009b).

These studies are particularly interesting in that although they all postulate significant neural elements in CoM braking, the demonstration of hysteresis in the current study suggests that there is also a sizeable interaction with the dynamics of the task (Getchell and Whitall, 2004). Orderly changes in walking speed and/or step length appear to provide the context for shaping central set mechanisms that takes into account external conditions and constraints to produce the desired behavior (Horak et al., 1989; Hayes et al., 1998; Chong et al., 1999a,b, 2000).

Demonstrating the presence of hysteresis is also a strong evidence that we are studying a dynamical system of walking gait mechanisms which is influenced by how it is expressed previously. The entrainment of the walking gait pattern which arises from feedback (somatosensory inputs) and feedforward (gait pattern generators) sources (Kuo, 2002) at a given speed leads to the development of the neural central set (Taga et al., 1991). The neural set must be adjusted when a new speed is encountered. The presence of the hysteresis therefore implies a history effect, as if there is resistance of the dynamical system to change unless compelled to do so (Schöner and Kelso, 1988). The new behavioral set which the system has been coerced to change carries with it the baggage of the previous input influences and output responses, so to speak.

## Author Contributions

All authors listed, have made substantial, direct and intellectual contribution to the work and approved it for publication.

## Conflict of Interest Statement

The authors declare that the research was conducted in the absence of any commercial or financial relationships that could be construed as a potential conflict of interest.
